# Conjunctival Involvement of T-Cell Lymphoma in a Patient with Mycosis Fungoides

**DOI:** 10.1155/2016/4786498

**Published:** 2016-02-18

**Authors:** Sultan S. Aldrees, Pablo Zoroquiain, Sarah A. Alghamdi, Patrick T. Logan, Sonia Callejo, Miguel N. Burnier

**Affiliations:** ^1^Henry C. Witelson Ocular Pathology Laboratory, McGill University, 1001 Boulevard Decarie, Montreal, QC, Canada H4A 3J1; ^2^Department of Ophthalmology, College of Medicine, King Saud University, P.O. Box 245, Riyadh 11411, Saudi Arabia; ^3^Department of Ophthalmology, McGill University, 5252 Boulevard de Maisonneuve Ouest, Montreal, QC, Canada H4A 3S5

## Abstract

*Background*. Ocular involvement in mycosis fungoides (MF) cases occurs in one-third of patients with the eyelid being the most frequent site affected; however, conjunctival involvement is rarely reported. Herein, we report a rare case of conjunctival involvement of MF.* Case Presentation*. A 66-year-old man who was previously diagnosed with MF in 2010 and was treated presented in 2014 complaining of foreign body sensation and redness in both eyes. Slit lamp examination of both eyes showed erythematous conjunctival growth that extended circumferentially. Physical examination revealed erythematous skin lesions on different body parts. Conjunctival biopsy was performed and revealed a dense, highly polymorphic lymphocytic population. The immunophenotype demonstrated a neoplastic T-cell origin consistent with MF. A diagnosis of conjunctival involvement by MF was made. The conjunctiva was treated with radiotherapy resulting in tumor regression. There were no recurrences at the 6-month follow-up.* Conclusion*. T-cell lymphoma should be considered in patients with a history of MF presenting with conjunctival and skin lesions.

## 1. Background

Lymphoproliferative tumors of the eye and ocular adnexa are usually non-Hodgkin's B-cell lymphomas with the marginal zone type (mucosa-associated lymphoid tissue (MALT)) being the most common one [1, 2]. T-cell lymphoma of the ocular adnexa is rare, and when it occurs it is usually a manifestation of systemic T-cell lymphoma or represents a progression of mycosis fungoides (MF) on the skin [2]. However, only about 2% of people with cutaneous T-cell lymphoma have ophthalmic manifestations with the eyelid being the most common site to be affected [3]. To the best of our knowledge, T-cell lymphoma of the conjunctiva in a patient with MF has only been reported four times in the English literature (Table 1) [4–6]. We hereby report a rare case of conjunctival involvement of T-cell lymphoma in a patient with MF. Moreover, this is the first reported case with a comprehensive immunohistochemical analysis.

## 2. Case Presentation

**Figure 1:**
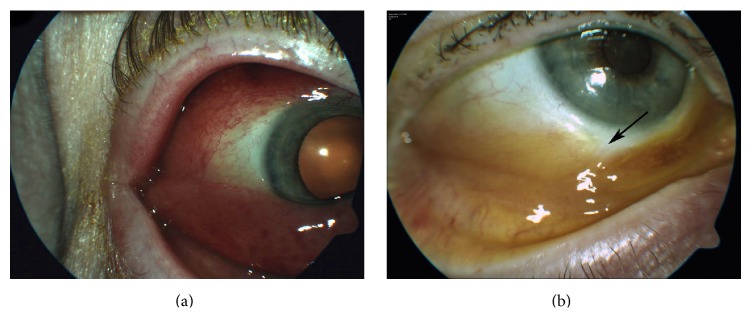
Slit lamp images of the right eye. (a) Erythematous flesh-like tumor affecting most of the conjunctiva, which is more prominent in the inferior location. (b) After treatment, there was significant lesion regression. Note the scar at the biopsy site (arrow).

**Figure 2:**
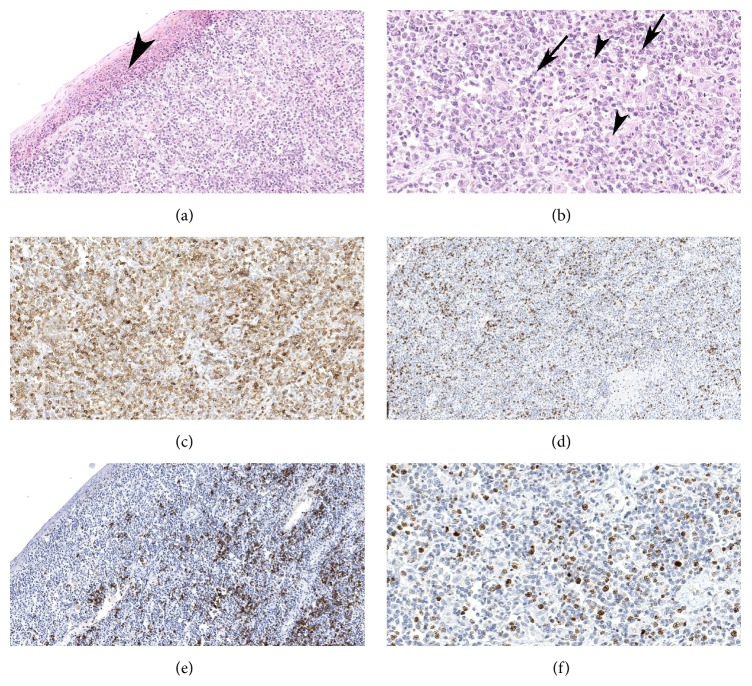
Histopathological analysis of the biopsy. (a) The conjunctiva was extensively infiltrated by atypical lymphocytes with epidermotropism (arrowhead; Hematoxylin and Eosin [H and E], 200x). (b) At higher magnification, the tumor showed atypical small to medium sized lymphocytes with irregular nuclei and clumped chromatin (arrow). Macrophages and reactive lymphocytes were also seen (H and E, 400x). (c) The neoplastic cells were positive for CD3 (200x). (d) Decreased coexpression by the neoplastic lymphocytes was noticed (100x). (e) CD20 was only expressed by the scattered reactive B-cells (100x). (f) Proliferative index estimated by nuclear positivity for KI-67 antigen was approximately 20% (400x).

A 66-year-old man with a known history of hypertension, hypercholesterolemia, and MF (without extracutaneous manifestations) that was diagnosed in 2010 and treated with total skin electron beam irradiation as well as boost treatments to numerous areas presented to the clinic in 2014 with foreign body sensation and redness in both eyes that had persisted for few months. This foreign body sensation was more in the right eye than in the left eye and was not associated with any tearing or decreased vision. Visual acuity at presentation was 20/40 in both eyes. Slit lamp examination of the right eye showed rubbery erythematous growth of the conjunctiva extending circumferentially around the globe; however, it was more prominent in the inferior part of the conjunctiva (Figures 1(a) and 1(b)). The cornea was not involved. Examination of the left eye showed similar findings but with lesser degree. At this stage, a clinical diagnosis of B-cell lymphoma (MALT type) involving the conjunctiva was made; however, the patient gave a history of several similar skin lesions to the ones he had in 2010 that started to appear around the same time of the eye complaint. Physical examination of the body revealed erythematous skin lesions mainly on the back, between the buttocks, and left medial superior thigh. A progression of the disease to involve the conjunctiva was then suspected and a conjunctival biopsy was performed. Microscopically, the conjunctival specimen showed an infiltration of neoplastic lymphocytes; some of them were large cells with large and irregular nuclei. Overall, the neoplastic population was pleomorphic and included cells with small, medium, and large cribriform nuclei as well as blast cells with prominent nuclei and mitotic figures. The immunohistochemical panel (Figures 2(a)–2(f)) was positive for T-cell markers, including CD2, CD3, CD4, and CD5. Conversely, the tumor cells did not express CD7 or CD8 indicating the malignant nature of the cells and favoring the diagnosis of extracutaneous MF. CD20, CD10, and CD23 (B-cell markers), anaplastic lymphoma kinase (ALK), and CD30 were all negative. Ki 67 was positive in 20% of the neoplastic cells. A diagnosis of conjunctival involvement by mycosis fungoides was subsequently made. Magnetic resonance imaging of the brain showed thickening of the anterior periocular tissue in both eyes without orbital extension. The patient received low dose radiotherapy of 4 grays (Gy) in two fractions and showed significant tumor regression with good tolerability. After 6 months of follow-up, no tumor recurrence was noted. All data accumulation was in accordance with Canada and Province of Quebec legislation and the tenets of the Declaration of Helsinki.

## 3. Discussion

MF is the most common type of primary cutaneous T-cell lymphoma and is usually defined as peripheral epidermotropic cutaneous T-cell lymphoma with small and medium-sized infiltrating T-cells with cerebriform nuclei [8]. The clinical picture of the disease follows a specific stepwise pattern from patches to plaques and mushroom-shaped tumors [9]. Visceral and lymph node involvement is seen at late stages of the disease and usually involves the lungs, spleen, and liver [10, 11]. Ocular involvement can be found in up to one-third of cases with the eyelid being the most common involved site [3, 4, 12, 13]. The most prevalent ocular findings in MF cases are eyelid tumor, cicatricial eyelid ectropion, eyelid thickening, blepharitis, and corneal abnormalities [3]. However, conjunctival involvement is extremely rare [4–6]. To the best of our knowledge, conjunctival involvement in a patient with MF has only been reported four times in the English literature (Table 1). Moreover, this is the first report with extensive immunohistochemical analysis of such a case.

The diagnosis of cutaneous T-cell lymphoma may be challenging, especially during early stages because of the overlapping features with inflammatory dermatosis [9]. However, immunophenotyping serves as an important tool in the evaluation of lymphoproliferative diseases. Microscopically, MF is usually characterized by proliferating T-lymphocytes that mostly express CD2, CD3, CD4, and CD5 with less expression of CD8 [9, 14]. Moreover, CD7 will be less expressed in the malignant T-cells and can be used as a marker to distinguish between inflammatory and neoplastic T-cell conditions [9]. This known immunoprofile is similar to our case in which we found high expression of CD3 and CD4 compared to CD7 and CD8. CD10 is a marker of follicular center B-cell lymphoma. However, it can be expressed in rare cases of MF with follicular helper T-cells [15]. In our case the expression of CD10 was only evident in the reactive B-cells. This immunohistochemical panel performed herein was similar to the one administered at the time of the diagnosis of MF in 2010.

The differential diagnosis of T-cell lymphoma affecting the eye and the periocular adnexa is varying and can include peripheral T-cell lymphoma, not otherwise specified (PTCL-NOS), anaplastic large T-cell lymphoma (T-ALCL), and T/natural killer (T/NK) lymphoma of the nasal type [16]. PTCL-NOS usually shows a mixture of small and large atypical cells that express T-cell markers (CD2, CD3, CD5, and CD7) in variable degrees [17]. On the other hand, T-ALCL usually shows large cells with abundant cytoplasm and characteristic cells with kidney shaped nucleus surrounded by eosinophilic cytoplasm [18]. This subset of T-cell lymphoma often expresses anaplastic lymphoma kinase (ALK) in cases of ALK + ALCL and usually has a strong positivity for CD30 [16, 19]. T/NK lymphoma is characterized by an angiocentric pattern where small to medium tumor cells obstruct the vessels and cause necrosis. These tumor cells show positivity for CD2, CD56, EBER-1, and cytoplasmic CD3 epsilon [20].

The treatment of conjunctival involvement of MF varies throughout the literature (Table 1). Stenson and Ramsy report using topical steroids and radiotherapy with a good initial response, but the patient died from complications of the skin tumors [4]. In 1985, O'Day et al. [5] reported treating a patient's skin lesions with psoralen long-wave ultraviolet (PUVA), but since she was wearing UV blocking glasses during treatment she developed a relapse in an area of the conjunctiva that was not exposed to PUVA, the so-called sanctuary site. This relapse site was then treated by superficial radiotherapy. Rubegni et al. reported surgical excision with low doses of recombinant interferon- (INF-) *α*2*α* plus etretinate as their treatment of choice in order to avoid cataract formation associated with local radiotherapy [6]. In the present case, our patient was treated by superficial radiation with no documented cataract formation. Moreover, there was no relapse of the tumor after 6 months of follow-up.

## 4. Conclusion

In conclusion, herein we report a rare case of conjunctival involvement in a patient diagnosed with MF. Full history and physical examination are key factors for the correct differential diagnosis. Although B-cell lymphoma is more common, T-cell lymphoma should be considered in patients presenting with a conjunctival lesion and a history of MF. Immunohistochemistry is a crucial tool for proper diagnosis and classification of lymphoproliferative diseases of the eye and ocular adnexa.

## Figures and Tables

**Table 1:** Cases of T-cell lymphoma of the conjunctiva in patients with mycosis fungoides in the English literature.

Author	Publication year	Gender	Age	Ocular structures involved	Method of treatment	Follow-up
Fradkin et al. [7]	1969	F	42	Left caruncle	Radiotherapy with strontium-90	Not reported

Stenson and Ramsy [4]	1981	F	62	Conjunctiva and eyelid	Topical steroids with radiotherapy	Good initial eye response; patient died from skin cancer complications

O'Day et al. [5]	1985	F	40	Conjunctiva	Radiotherapy	Not reported

Rubegni et al. [6]	1997	M	34	Conjunctiva	Surgery with low dose rINF-*α*2*α* plus etretinate	No relapse of skin or eye tumor after 1-year follow-up

Present report	2015	M	66	Conjunctiva	Radiotherapy	No relapse up to 6 months of follow-up

F: female; M: male.

## Abbreviations

MF: Mycosis fungoides
MALT: Mucosa-associated lymphoid tissue
PUVA: Psoralen long-wave ultraviolet
Gy: Gray.
